# Performance, Safety and Usability of a Third-Generation Continuous Glucose Monitoring System in a Pediatric Population: Results From a Prospective, Multicenter Study

**DOI:** 10.1177/19322968261463536

**Published:** 2026-07-19

**Authors:** Fengyun Wang, Frank Flacke, Shuxia Ding, Wenxian Oyang, Haiying Wu

**Affiliations:** 1 Children’s Hospital of Soochow University, Suzhou, China; 2 Flacke Consulting GmbH, Mainz, Germany; 3 Sinocare Inc., Changsha, China; 4 Women and Children’s Hospital of Ningbo University, China; 5 Hunan Children’s Hospital, Changsha, China

**Keywords:** pediatrics, continuous glucose monitor, mean absolute relative difference (MARD), sensor accuracy, wear life

## Abstract

**Background:**

A proprietary continuous glucose monitoring (CGM) system that uses third-generation sensor technology has previously been shown to exhibit stable, single-digit mean absolute relative difference (MARD) and 15-day wear life in adults with diabetes.

**Method:**

This was a prospective, single-arm study of pediatric participants aged 2 to 17 years that were enrolled at 3 clinical centers. Participants wore 2 sensors, one on each side of the abdomen, and were randomized to assessment of device performance on days 1 and 2, days 7 to 9 or days 15 and 16. Device performance across a range of metrics was assessed by comparison with venous blood glucose values obtained using a laboratory reference device. Per-protocol results are reported for the sensor with the inferior MARD.

**Results:**

The per-protocol set comprised 75 participants, of whom 16 were aged 2 to 5 years. The overall MARD was 8.89%, and MARDs at days 1 and 2, 7 to 9 and 15 and 16 were 8.65%, 8.70% and 9.30%, respectively. DTS error grid analyses showed that 100.0% of data pairs fell in clinically acceptable zones A+B. True alarm rates for hypoglycemia and hyperglycemia were high, at 98.6% and 98.8%, respectively. Mean sensor wear life was 14.4 days, and participant/guardian assessments of sensor usability revealed high satisfaction with respect to system assembly, sensor insertion and overall comfort. No device-related or skin-related adverse events were reported.

**Conclusions:**

In a pediatric population, the novel CGM system demonstrated accurate and stable performance across its 15-day wear life and showed high usability.

## Introduction

Continuous glucose monitoring (CGM) systems that use a minimally invasive approach to measure glucose in interstitial fluid have led to significant advances in the management of diabetes. As a result, management guidelines now recommend the integration of CGM with multiple daily insulin injections or to guide continuous insulin infusion as the standard of care for outpatient diabetes management.^1-4^ The American Diabetes Association guidelines also recommend CGM for patients on basal insulin.^2^ These clinical advances have been enabled by developments in sensor technology, which have afforded increased sensor accuracy, reliability and wear times.^5^

The iCan sensor is a factory-calibrated 15-day CGM sensor that uses third-generation, direct electron-transfer technology with modified glucose dehydrogenase (GDH) with a flavin adenine dinucleotide (FAD) cofactor as the electron acceptor.^6^ This novel sensor has been tested in a total of 507 people with diabetes in trials conducted at clinical centers in the United States, Europe and Asia, with a total of 295 adults^7-9^ and 212 children aged 2 to 17 years enrolled.^10,11^ Here, we report the results on the performance, safety and usability of the sensor in a pediatric population aged 2 to 17 years.

## Methods

### Study Design

This was a prospective, multicenter, single-arm study that was conducted complying to Good Clinical Practice and the declaration of Helsinki at 3 investigational sites in China (registered on ClinicalTrials.gov as NCT06570551). Approval was obtained from the ethics committees of each of the participating institutions, and written informed consent was obtained from each subject and/or his/her guardian.

The main study inclusion criteria were: (1) age 2 to 17 years; (2) clinical diagnosis of diabetes; (3) willingness to wear the device continuously for 15 days and conduct blood collection for blood glucose testing in accordance with the study requirements; (4) body weight ≥10.0 kg. The main exclusion criteria were: (1) need to undergo magnetic resonance imaging (MRI) during the study; (2) diffuse subcutaneous nodules; (3) acute complications of diabetes; (4) abnormal coagulation function (activated partial thromboplastin time and/or prothrombin time >1.5× the upper limit of normal or less than the upper limit of normal).

Participants wore 2 sensors, one on each side of the abdomen, and the device performance was assessed by comparison with a point of care, enzymatic-amperometric glucoseoxidase-based, calibratable laboratory reference device (Biosen C-Line glucose and lactate measuring instrument, EKF-diagnostic GmbH). For data consistency, the sensor placed on the right side of the abdomen with the poorer overall performance was chosen for data reporting. Subjects were randomly allocated to assessment of device performance on days 1 and 2 (Group A), days 7 to 9 (Group B) or days 15 and 16 (Group C). During these intervention days, the subjects were provided with a meal to induce glucose changes, but no intentional delay or increase of insulin doses was applicable where applied. On day 16, the devices were removed, and subjects were asked to complete a feedback questionnaire (completed by the subject’s guardian in case of children aged ≤6 years).

### Statistical Analysis

In accordance with the latest requirements from the Center for Medical Device Evaluation of the National Medical Products Administration, the study planned to enroll a total of 78 subjects, with no less than 60 subjects aged 6 to 17 years and at least 15 subjects aged 2 to 5 years. The full analysis set (FAS) included all cases that expressed the intention to accept CGM and sign the informed consent form, had used the investigational device and had post-CGM evaluation data. The per-protocol set (PPS) was a subset of the FAS and consisted of subjects having worn sensors as specified in the study protocol, with available observational data on the primary endpoints and no major protocol violation. The safety set comprised all subjects who applied the investigational device at least once.

The primary endpoints were the proportion of CGM values that fell within ±20% or ±20 mg/dL of the reference values (agreement rate [AR]); the proportion of measurement points falling within zones A+B according to the consensus error grid (C-EGA)^12^ and mean absolute relative difference (MARD). A post-hoc analysis evaluated the proportion of measurement points falling within zones A+B according to the Diabetes Technology Society error grid (DTS EG).^13^ Secondary endpoints included: (1) true notification rates of hypoglycemia and hyperglycemia; (2) sensor stability; (3) sensor repeatability; (4) sensor life and (5) product usability. Statistical analyses were performed using SAS 9.4 (SAS).

## Results

**Figure 1.**
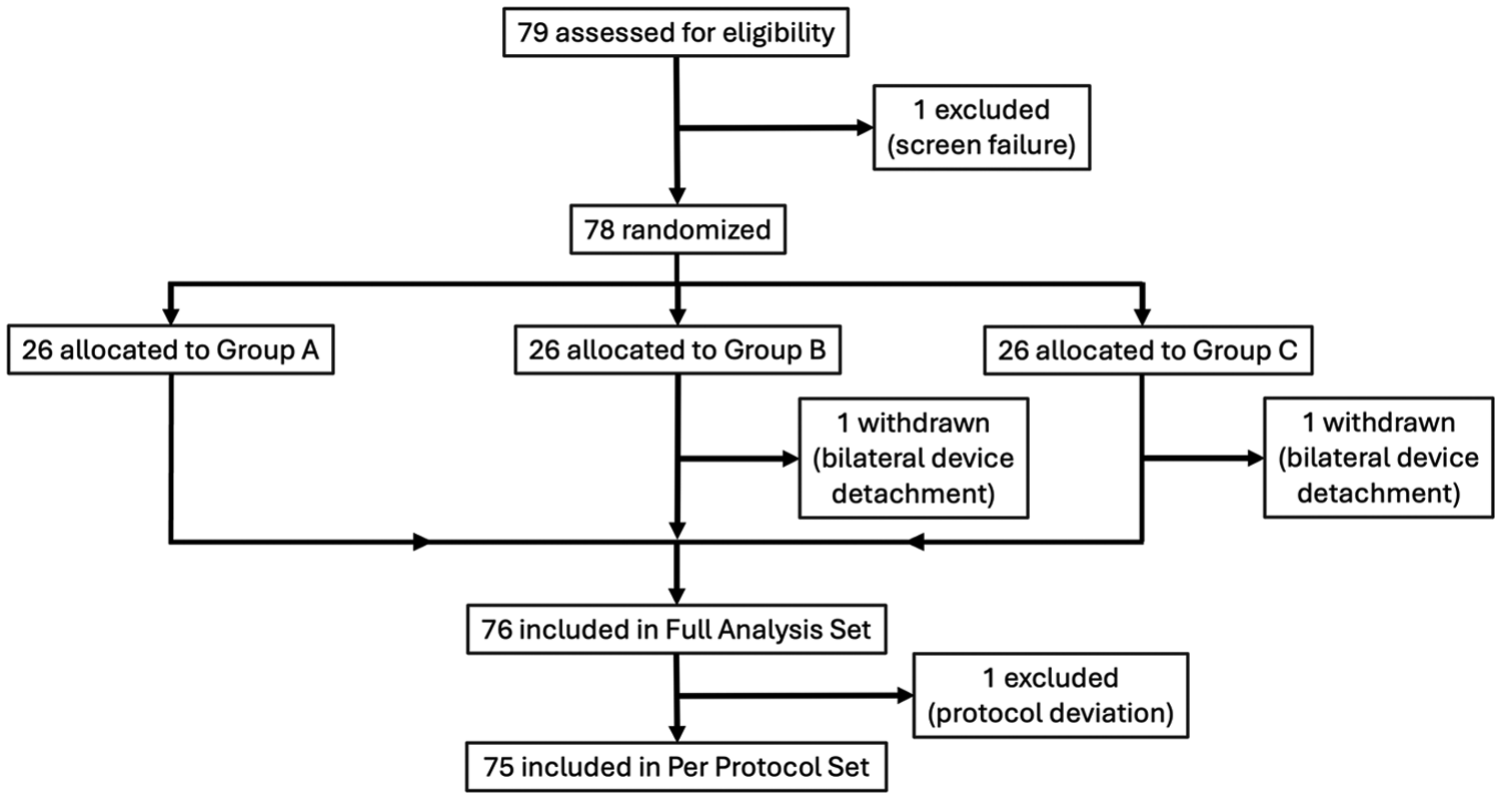
Study flowchart.

Participant flow is summarized in Figure 1. A total of 78 subjects were enrolled, and ultimately 76 subjects completed the clinical trial. The FAS consisted of 76 participants, of whom 16 were aged 2 to 5 years, and 60 were aged 6 to 17 years. Most participants had type 1 diabetes (86.8%). Baseline data are summarized in Table 1.

**Table 1.** Baseline Data (Full Analysis Set).

Variable	Age 6-17 years (N = 60)	Age 2-5 years (N = 16)
Gender, male/female (%)	33/27 (55/45)	6/10 (37.5/62.5)
Diabetes type, n (%)		
Type 1	52 (86.7)	14 (87.5)
Other special	1 (1.7)	0 (0)
Unspecified	7 (11.7)	2 (12.5)
Age (years), mean ± SD	11.5 ± 3.5	4.6 ± 1.0
Height (cm), mean ± SD	147.5 ± 18.8	107.0 ± 9.0
Weight (kg), mean ± SD	43.1 ± 17.1	18.8 ± 4.0
Duration of diabetes (days), mean ± SD	1174.8 ± 764.20	606.8 ± 456.66

### Primary Endpoints

**Figure 2.**
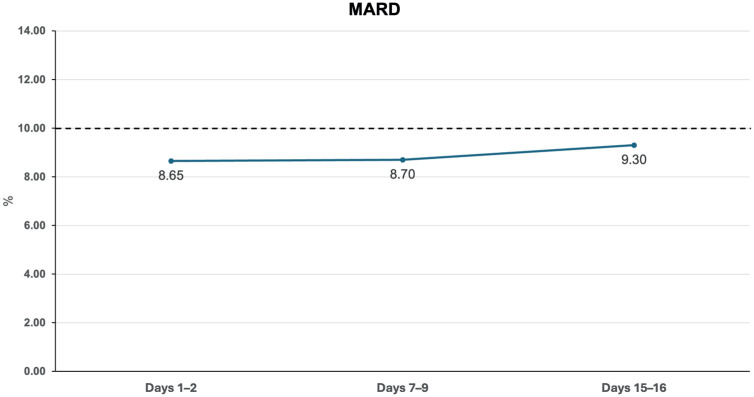
MARD stability over wear life. The horizontal dashed line indicates the 10% MARD threshold for eligibility for replacement of fingerstick glucose tests (nonadjunctive use).

**Figure 3.**
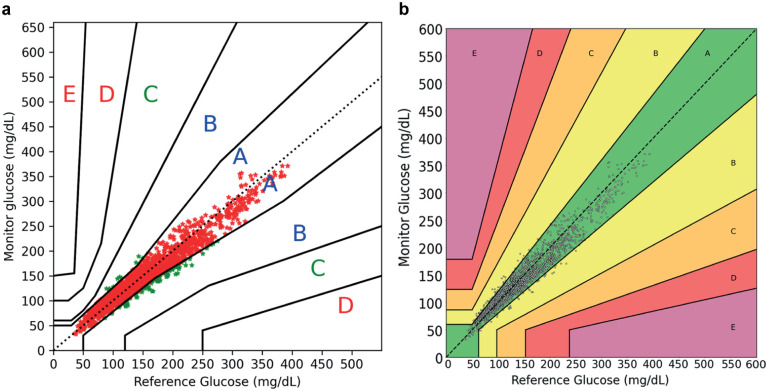
Error grid analyses for subjects with type 1 diabetes (per-protocol sets). (a) Consensus error grid. (b) Diabetes Technology Society error grid.

Per-protocol analysis demonstrated strong sensor performance with an overall 20/20% AR of 95.96% (95% confidence interval: 95.69%-96.23%). Based on 1879 data pairs, the overall MARD was 8.89%, with stable MARD results across a 15-day wear time (days 1-2, 8.65%; days 7-9, 8.70%; days 15-16, 9.30%) (Figure 2). The device demonstrated single-digit MARD/MAD across multiple different glucose levels (Table 2). The C-EGA analysis and a post-hoc DTS EG analysis both showed that 100.0% of data pairs fell in clinically acceptable zones A+B (Table 3 and Figure 3).

**Table 2.** Sensor Accuracy Across Glucose Ranges: Per-Protocol Set.

EKF glucose concentration, mg/dL	% within 15/15%	% within 20/20%	% within 30/30%	% within 40/40%	MAD, mg/dL or MARD, %	No. of data pairs	No. of subjects
<70	93.33	99.17	100.00	100.00	7.26	120	34
70 to <180	86.78	95.17	99.60	100.00	8.55%	1241	72
180 to <250	83.01	96.94	100.00	100.00	9.41%	359	52
≤250	88.05	97.48	100.00	100.00	7.93%	159	19
Overall	86.59	95.96	99.73	100.00	8.89%	1879	72

**Table 3.** Error-Grid Data: Per-Protocol Set.

Zone	No. of data pairs	Proportion (%)	95% Confidence interval
Consensus error grid			
A	1798	95.7	94.67%-96.52%
B	81	4.3	3.48%-5.33%
C	0	0	0.0%-0.2%
C	0	0	0.0%-0.2%
E	0	0	0.0%-0.2%
A+B	1879	100.0	99.8%-100.0%
Diabetes Technology Society error grid			
A	1781	94.8	93.68%-95.75%
B	98	5.2	4.25%-6.32%
C	0	0	0.00%-0.2%
C	0	0	0.00%-0.2%
E	0	0	0.00%-0.2%
A+B	1879	100.0	99.8%-00.00%

### Secondary Endpoints

The sensor demonstrated reliable true notification rates for hypoglycemia and hyperglycemia. Of 146 alarms issued for hypoglycemia (80 mg/dL, 4.4 mmol/L), 144 were preceded or followed within 15 minutes by a reference glucose measurement above the threshold level. Of 248 alarms issued for hyperglycemia (200 mg/dL, 11.1 mmol/L), 245 met the criteria for a true alarm. Thus, the true alarm rates for hypoglycemia and hyperglycemia were 98.6% and 98.8%, respectively.

For the right abdomen, the 20/20% AR results with the reference value were 95.83% (95% CI: 95.47%-96.20%) at days 1 and 2, 97.34% (95% CI: 97.00%-97.67%) at days 7 to 9 and 94.76% (95% CI: 94.14%-95.38%) at days 15 and 16. Post-hoc analysis showed that mean sensor wear life was 14.41 days; 92.0% of right sensors in the PPS had a wear life of 15 days.

Regarding sensor insertion, 86.7% of the subjects reported no pain, 13.3% reported moderate pain and none reported severe pain (PPS). The insertion process was considered very convenient for 97.3% of participants, while it was considered generally acceptable for 2.7% of participants (PPS). Similarly, the assembly convenience of the glucose signal transmitter and glucose sensor was rated as very convenient in 97.3% of participants, and generally acceptable in 2.7% of participants. When evaluating the overall comfort of use during the 16-day study period, the device was reported as comfortable for 93.3% of participants, and acceptable in 6.7%.

### Safety

Of the 78 subjects in the safety set, 51 experienced 82 adverse events, of which 4 subjects received corrective treatment. Of note, there were no device-related adverse events or skin-related adverse events reported; most adverse events were attributable to expected events (Table 4). There was 1 serious adverse event of hypertension (unrelated to the study device).

**Table 4.** Adverse-Event Data (Safety Set; N = 78).

Adverse event category	No. of events	No. of subjects	Incidence (%)
Any adverse event	82	51	65.4
Serious adverse event	1	1	1.3
Adverse event requiring corrective treatment	5	4	5.1
Adverse event related to device	0	0	0
Adverse event associated with withdrawal from the study	0	0	0
Type of adverse event		n	%
Hyperglycemia	26	26	33.3
Hypoglycemia	51	51	65.4
Skin abnormality	0	0	0
Other	5	5	6.4

## Discussion

The glucose sensor that was evaluated in this pediatric study is a third-generation sensor that uses modified glucose dehydrogenase coupled with FAD as the electron acceptor.^6^ Based on glucose measurements at days 1 and 2, 8 and 9 and 15 and 16 of the study, the overall MARD was 8.89%, which is consistent with the overall MARD of 8.71% observed with the same system in a multicenter study of adults with diabetes.^7^ A sub-9% MARD for the device under study compares favorably with MARDs that have been reported for other contemporary CGM devices in pediatric populations. Previously reported MARDs are 9.7% for FreeStyle Libre 2 in subjects aged 4 to 17 years^14^; 10.0% and 8.6% with FreeStyle Libre 3 in subjects aged 4 to 5 years and 6 to 17 years, respectively^15^; 9.9% and 9.6% for Dexcom G6 in subjects aged 2 to 5 years and 6 to 17 years, respectively^16^; 9.0% for abdomen-placed Dexcom G7 in subjects aged 7 to 17 years^17^ and 11.6% for the Guardian 4 sensor in subjects aged 7 to 17 years.^18^ The MARD that was obtained in the current study was complemented by the findings that 96.0% of CGM glucose readings were within 20/20% AR of the reference value, and that 100% of data pairs in error grid analyses fell in clinically acceptable zones A+B in both analyzed error grids.

Moreover, the MARD showed good stability over time, reflecting the reliability of the device over the 15-day wear life. This contrasts with a drop-off in accuracy toward the end of sensor life that has been seen with older CGM technology; thus, in a study of FreeStyle Libre 2 in children aged 4 to 17 years, mean MARD was 8.0% at days 7 and 8, 9.7% at days 9 to 12 and 10.2% at days 13 and 14.^14^ In the present study, 92.0% of right sensors in the PPS had a wear life of 15 days. This compares favorably with the durability in children that has been reported for other devices, such as 75.7% sensor survival at 10 days for Dexcom G6^16^ and 78.1% sensor survival at 14 days for FreeStyle Libre 2.^14^

Prespecified secondary analyses also demonstrated good performance in terms of accuracy at different glucose concentrations and high rates of true alerts for hyperglycemia and hypoglycemia.

There were no device-related adverse events or skin-related adverse reported during the study, consistent with results obtained for the sensor in the study of adults with diabetes.^7^ One serious adverse event in the present study, diagnosed as hypertension with a severity of Grade 1 (CTCAE, v5.0), was deemed to be unrelated to the device. Another potential safety consideration is the reduced need for sensor changes over the course of a year compared to sensors with targeted wear life of 10 days or 14 days and therefore a reduction in the length of time spent with sensors that are running in, since the run-in period is usually about a day.

Certain strengths and limitations of this study should be mentioned. Our study included children as young as 2 years to provide performance data on a broad range of ages. In addition, we adopted a conservative approach for data analysis, focusing on the performance characteristics of the poorer performing sensor, that is, the sensor applied to the right abdomen. No experimental glucose manipulations were performed during the study; glucose variations were those occurring naturally because of meals, drinks or routine medication. Ethical requirements prevented the participation of each subject at each performance evaluation visit. Therefore, the potential for bias was minimized by random allocation of subjects across the 3 performance evaluation visits.

## Conclusion

Pivotal clinical trials of an innovative CGM system have enrolled more than 500 adult and pediatric participants with diabetes. In the current performance study in pediatric participants aged between 2 and 17 years, the novel CGM system demonstrated high overall accuracy and a single-digit MARD that is comparable to those of established systems across its 15-day wear time. This performance translates into high detection rates for both hypoglycemia and hyperglycemia. In addition, an outstanding average sensor wear life that is close to the intended wear time and no device-related adverse events – as already seen in an adult population – can be reported.

## References

[1] Limbert C Tinti D Malik F , et al. ISPAD clinical practice consensus guidelines 2022: the delivery of ambulatory diabetes care to children and adolescents with diabetes. Pediatr Diabetes. 2022;23(8):1243-1269. doi:10.1111/pedi.134172 36537530

[2] American Diabetes Association Professional Practice Committee. 7. Diabetes technology: standards of care in diabetes – 2024. Diabetes Care. 2024;47(Suppl 1):S126-S144. doi:10.2337/dc24-S007 PMC10725813 38078575

[3] Holt RIG DeVries JH Hess-Fischl A Hirsch IB Kirkman MS , et al. The management of type 1 diabetes in adults. A consensus report by the American Diabetes Association (ADA) and the European Association for the Study of Diabetes (EASD). Diabetologia. 2021;64(12):2609-2652. doi:10.1007/s00125-021-05568-3 34590174 PMC8481000

[4] Tauschmann M Forlenza G Hood K , et al. ISPAD clinical practice consensus guidelines 2022: diabetes technologies: glucose monitoring. Pediatr Diabetes. 2022;23(8):1390-1405. doi:10.1111/pedi.13451 36537528 PMC10107687

[5] Morales-Dopico L MacLeish SA. Expanding the horizon of continuous glucose monitoring into the future of pediatric medicine. Pediatr Res. 2024;96(6):1464-1474. doi:10.1038/s41390-024-03573-x 39306610 PMC11624137

[6] Ferri S Kojima K Sode K. Review of glucose oxidases and glucose dehydrogenases: a bird’s eye view of glucose sensing systems. J Diabetes Sci Technol. 2011;5(5):1068-1076. doi:10.1177/193229681100500507 22027299 PMC3208862

[7] Ji L Flacke F Gao F , et al. ICan CGM system—performance evaluation of a new CGM system with an innovative sensor technology. Diabetes. 2024;73(Suppl 1):987-P.

[8] ClinicalTrialsgov. NCT05806554. Accuracy and precision of the True Vie I3 continuous monitoring system: an open-label, multi-center trial. Accessed August 29, 2025. https://clinicaltrials.gov/study/NCT05806554.

[9] ClinicalTrialsgov. NCT05348928. A pilot study of the feasibility and accuracy of the TrueVie CGM system - A non-significant risk study. Accessed October 20, 2025. https://clinicaltrials.gov/study/NCT05348928?cond=continuous%20glucose%20monitoring&viewType=Table&rank=4

[10] Zheng J Fei J Gao F , et al. User reported outcome and alert performance of the iCan CGM system in a pediatric population. Diabetologica. 2025;68(Suppl 1):S489. doi:10.1007/s00125-025-06497-1

[11] ClinicalTrialsgov. NCT05908448. The True Vie I3 continuous glucose monitoring system in pediatric patients with type 1 diabetes. Accessed August 29, 2025. https://clinicaltrials.gov/study/NCT05908448

[12] Parkes JL Slatin SL Pardo S Ginsberg BH. A new consensus error grid to evaluate the clinical significance of inaccuracies in the measurement of blood glucose. Diabetes Care. 2000;23(8):1143-1148. doi:10.2337/diacare.23.8.1143 10937512

[13] Klonoff DC Freckmann G Pleus S , et al. The diabetes technology society error grid and trend accuracy matrix for glucose monitors. J Diabetes Sci Technol. 2024;18(6):1346-1361. doi:10.1177/19322968241275701 39369312 PMC11531029

[14] Alva S Bailey T Brazg R , et al. Accuracy of a 14-day factory-calibrated continuous glucose monitoring system with advanced algorithm in pediatric and adult population with diabetes. J Diabetes Sci Technol. 2022;16(1):70-77. doi:10.1177/1932296820958754 32954812 PMC8875061

[15] Alva S Brazg R Castorino K Kipnes M Liljenquist DR Liu H. Accuracy of the third generation of a 14-day continuous glucose monitoring system. Diabetes Ther. 2023;14(4):767-776. doi:10.1007/s13300-023-01385-6 36877403 PMC10064376

[16] Dexcom. Dexcom G6 continuous glucose monitoring system user guide. LBL014003 Rev 012 MT23976. Accessed July 17, 2025.https://s3-us-west-2.amazonaws.com/dexcompdf/G6-CGM-Users-Guide.pdf

[17] Laffel LM Bailey TS Christiansen MP Reid JL Beck SE. Accuracy of a seventh-generation continuous glucose monitoring system in children and adolescents with Type I diabetes. J Diabetes Sci Technol. 2023;17(4):962-967. doi:10.1177/19322968221091816 35466707 PMC10347986

[18] Medtronic. MiniMed™ 780G system with Guardian™ 4 sensor. System user guide. Accessed July 17, 2025. https://www.medtronicdiabetes.com/sites/default/files/library/download-library/user-guides/MiniMed-780G-system-user-guide-with-Guardian-4-sensor.pdf

